# Sex differences in telomere length, lifespan, and embryonic dyskerin levels

**DOI:** 10.1111/acel.13614

**Published:** 2022-04-20

**Authors:** Peter M. Lansdorp

**Affiliations:** ^1^ Terry Fox Laboratory BC Cancer Agency Vancouver BC Canada; ^2^ Department of Medical Genetics University of British Columbia Vancouver BC Canada

**Keywords:** cardiovascular disease, *DKC1*, dyskerin, immune disorders, lifespan, sex differences, telomerase, telomere length, X chromosome inactivation

## Abstract

Telomerase levels in most human cells are insufficient to prevent loss of telomeric DNA with each replication cycle. The resulting “Hayflick” limit may have allowed lifespan to increase by suppressing the development of tumors early in life be it at the expense of compromised cellular responses late in life. At any given age, the average telomere length in leukocytes shows considerably variation between individuals with females having, on average, longer telomeres than males. Sex differences in average telomere length are already present at birth and correspond to reported differences in the average life expectancy between the sexes. Levels of telomerase RNA and dyskerin, encoded by *DKC1*, are known to limit telomerase activity in embryonic stem cells. X‐linked *DKC1* is expressed from both alleles in female embryo cells and higher levels of dyskerin and telomerase could elongate telomeres prior to embryo implantation. The hypothesis that embryonic telomerase levels set the stage for the sex differences in telomere length and lifespan deserves further study.

## Graphical abstract

The telomere length in leukocytes declines with age. Short telomeres are a risk factor for cardiovascular and infectious diseases. Women have, on average, longer telomeres than males, a difference that is already present at birth. Here it is proposed that this difference originates during embryonic development as a result of bi‐allelic expression of X‐linked *DKC1* encoding dyskerin, a major component of the telomerase enzyme.

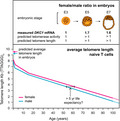

## 1 INTRODUCTION

Mutations in genes that provide a selective advantage early in life often have pleiotropic adverse effects late in life (Byars & Voskarides, 2020; Williams, 1957) and the genetic changes that suppress telomerase activity in somatic cells of long‐lived animals to limit their replication potential are no exception (Lansdorp, 2022). Limits to cell proliferation late in life are expected to impact cells of the immune system and cardiovascular system in particular. Of all leukocytes, NK cells and memory T cells show the most rapid decline in telomere length with age (Aubert et al., 2012) and replicative exhaustion of these and other immune cells is likely to impair immune responses in the elderly. Indeed, short telomeres have been linked to adverse COVID‐19 outcomes, independent of known risk factors for COVID including age (Wang et al., 2021). Short telomeres in leukocytes have also been associated with cardiovascular disease (Scheller Madrid et al., 2016; Xu et al., 2020) and telomere erosion in endothelial cells has been linked to vascular pathology (Chang & Harley, 1995). Infectious diseases and cardiovascular diseases are leading causes of death, and a link between telomere length and age‐related mortality is supported by several studies (Cawthon et al., 2003; Codd et al., 2021).

**FIGURE 1:**
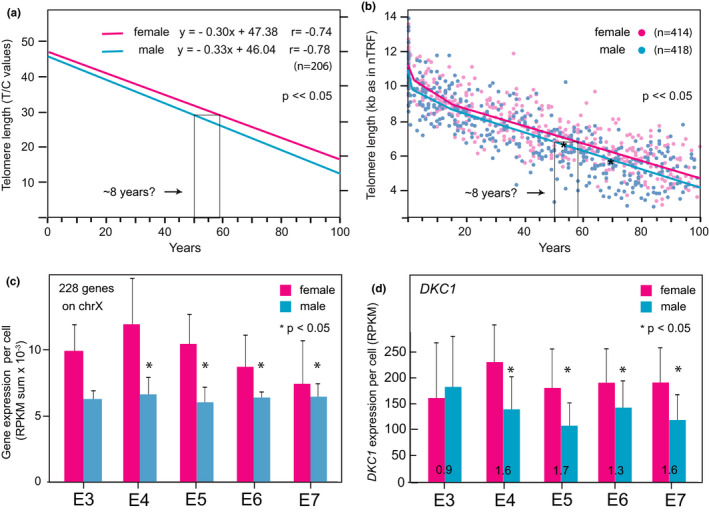
Sex differences in average telomere length, lifespan and embryonic dyskerin levels. (a, b) The average telomere length in T lymphocytes from males and females as reported in two independent studies (a) Quantitative fluorescence in situ hybridization was used to measure the ratio of fluorescence signals, expressed as T/C values, derived from fluorescent probes specific for resp. telomere, and centromere repeats on Chr 2 in metaphase cells from T‐cell cultures of 205 normal individuals in different age groups and sexes. See (Mayer et al., 2006) for details, reproduced with permission of S. Karger AG, Basel. (b) The telomere length in naive T cells from females (*n* = 414) and males (*n* = 418) and a male volunteer (asterikses: two different time points) was measured by flow FISH (Aubert et al., 2012). Note the large variation in average telomere length at any given age. Based on the difference in average telomere length in cultured (a) or circulating naive T cells (b), females are on average 8 years “younger” than males. (c, d) Delayed X chromosome inactivation results in bi‐allelic expression of most X chromosome genes in female embryo cells (c) including *DKC1* (d) prior to embryo implantation (Figure and data modified from (Petropoulos et al., 2016). (c) gene expression of 228 highly expressed X chromosome genes in single embryo cells; (d) expression of *DKC1*. Mean and standard deviation of normalized gene expression in single cells at indicated days 3–7 of embryonic development (E3–E7) expressed as reads per kilobase of transcript per million mapped reads (RPKM). The ratio of reads mapping to *DKC1* in female compared to male embryo cells is inserted in the bar graphs in (d). See (Petropoulos et al., 2016) for details

Most studies agree that the average leukocyte telomere length is between 0.1 and 0.3 kb longer in females than in males (Gardner et al., 2014). The telomere length in umbilical cord blood leukocytes was found to be longer in females compared with males by Q‐FISH (Mayer et al., 2006), flow FISH (Aubert et al., 2012), and by telomere restriction fragment (TRF) analysis (Factor‐Litvak et al., 2016) (Table 1 and Figure 1a,b). The rate of telomere attrition in adult human leukocytes was measured by TRF is 26 bp/year (Daniali et al., 2013), whereas by flow FISH, telomeric DNA is lost in adult lymphocytes at a rate of 43 bp/year (Aubert et al., 2012). By comparing the average telomere length and telomere attrition rate in adults with the average telomere length at birth, sex differences in telomere length correspond to a difference in average life expectancy between 5 and 8 years (Table 1 and Figure 1a,b), in close agreement with reported sex differences in average lifespan (Baum et al., 2021).

**TABLE 1:** Average telomere length in different umbilical cord blood cells is longer in female compared to male newborns

Technique (year)	Cell type	Location	TL female (*n*)	TL male (*n*)	Δ TL	Δ lifespan
Q‐FISH T‐C 2006 (Mayer et al., 2006)	Cultured T cells blood	Germany	12.03 kb (53)	11.81 kb (55)	0.22 kb	5.1 years
Flow FISH 2012 (Aubert et al., 2012)	Naive T cells blood	Canada	11.24 kb (29)	10.92 kb (29)	0.32 kb	7.4 years
TRF 2016 (Factor‐Litvak et al., 2016)	Leukocytes	USA	9.58 kb (216)	9.44 kb (274)	0.14 kb	5.3 years

Note

Results from three independent studies using three different techniques. Assuming a telomere attrition rate in cells from adults of around 26 bp/year for leukocytes (Factor‐Litvak et al., 2016) and 43 bp for lymphocytes (Aubert et al., 2012), telomere length differences at birth could account in principle for a sex difference in life expectancy between 5.1 and 7.4 years.

Currently, the sex difference in telomere length at birth is unexplained. On average, boys are typically heavier at birth than girls (Jelenkovic et al., 2018) and the male conceptus seems to grow not only more, but also earlier than the female (de Zegher et al., 1999). The telomere length in leukocytes declines most rapidly in the first few years of life in support of a mitotic clock ticking in blood forming stem cells and lymphocytes (reviewed in Lansdorp, 2022). In view of these observations, both gestational age and birth weight are expected to correlate with the telomere length in neonatal leukocytes. Such a correlation was indeed found in a small study (Sibert et al., 2021) but not in the large study that reported sex differences in leukocyte telomere length at birth (Table 1, Factor‐Litvak et al., 2016). Further studies of the telomere length in different pre‐ and post‐natal cells are needed to clarify the role of accumulated cell divisions in the sex‐specific difference in telomere length shown in Table 1. Given the shortcomings of all current telomere length measurements (Lansdorp, 2022), such studies should aim to measure the average as well as the distribution of telomere length values in cells.

Sex hormones provide another possible explanation for the sex differences in telomere length. Androgens are known to benefit patients with telomere biology disorders (Townsley et al., 2016), and the rate of telomere attrition in adult leukocytes is slightly higher in males than females (Figure 1a,b). Among other explanations, these observations could reflect the effect of sex hormones on *TERT* expression (Calado et al., 2009). Differences in the type and level of hormones secreted by the fetus and the placenta could also contribute to sex differences in average telomere length at birth. Alternatively, or in addition, differences in the expression of telomerase during early embryogenesis could also play a role. In a study of gene expression in single cells from human embryos, it was found that many X chromosome genes maintain bi‐allelic expression prior to embryo implantation and lineage specification (Petropoulos et al., 2016). During this time males, with only one copy of the X chromosome, express lower levels of X chromosome genes including the *DKC1* gene that encodes the dyskerin protein (Figure 1c,d and Table 2). Telomerase RNA is sandwiched between two copies of dyskerin in the telomerase holoenzyme (Ghanim et al., 2021) and dyskerin is critical for folding and stabilizing primary telomerase RNA transcripts, telomerase assembly, and telomerase activity (Wong & Collins, 2006). Whereas in most somatic cells, *TERT* expression is limiting telomerase levels, telomerase RNA is limiting the telomerase activity in embryonic stem cells (Chiba et al., 2015). Higher dyskerin levels in female embryo cells could increase telomerase levels by increasing the capture efficiency and stability of telomerase RNA prior to assembly with TERT, producing higher levels of active telomerase. Reduced telomerase levels were found in murine cells with reduced dyskerin levels (Ruggero et al., 2003) and allelic differences in *DKC1* are a plausible explanation for the reported X‐linked inheritance of telomere length in humans (Nawrot et al., 2004). Higher telomerase activity in female embryo cells before the random inactivation of one *DKC1* allele could result in longer telomeres in female compared with male embryos prior to embryo implantation. Longer telomeres in female embryo cells could explain why leukocytes from females at birth and throughout life have, on average, longer telomeres. Compared with males, longer telomeres in stem cells, endothelial cells, and lymphocytes from females could enable additional cell divisions before cells with critically short telomeres undergo either apoptosis or replicative senescence.

**TABLE 2:** Expression of selected genes in female and male human pre‐implantation embryos at Days 3–5 of development (E3–E5) prior to lineage specification (data from Petropoulos et al., 2016)

	E3		E4		E5	
	Female	Male	Female	Male	Female	Male
*DKC1*	185	162	232	142	193	112
*TERC*	4.0	2.7	2.4	2.1	3.7	1.7
*TERT*	0.2	0.2	4	4	4	4
*ZSCAN4*	1465	1369	30	37	0	2
*SOX2*	9	6	22	24	13	11
*POU5F1*	7	11	95	109	104	109
*KLF4*	12	18	92	89	129	86
*MYC*	819	709	103	173	108	64
*NANOG*	7	9	4	7	9	4
*TERF1*	3	2	4	3	3	4
*TERF2*	5	9	6	6	7	5
*POT1*	9	9	5	6	2	6
*CTC1*	1	1	1	1	1	2
*TINF2*	23	17	9	10	14	7
*TPP1*	0	1	1	1	4	6
*RIF1*	36	33	26	25	14	12
*RTEL1*	2	4	15	12	10	12

Note

Results are expressed as reads per kilobase of transcript per million mapped reads (RPKM). Genes on the X chromosome such as *DKC1* show bi‐allelic expression in female cells and, as a result, are expressed at higher levels compared with male cells (see also Figure 1d). Whereas *DKC1* transcripts were detected in every cell, *TERC* transcripts were only detected in a minority of cells (in 37/171 female and 51/206 male cells at E5). Selected other genes are shown for reference.

The relationships between sex, telomere shortening, and lifespan found in humans are not ubiquitous throughout the animal kingdom (Barrett & Richardson, 2011). The telomere length in several tissues from mice (*Mus spretus*) was found to be longer in females compared with males (Coviello‐McLaughlin & Prowse, 1997). In general, the mouse model has been extremely useful to identify genes that regulate telomere length (Ding et al., 2004). However, possible correlations between lifespan and telomere length are only expected in long‐lived species in which telomerase activity is suppressed to limit the replication potential in somatic cells and suppress tumor growth (Lansdorp, 2022). To test the hypothesis that embryonic dyskerin and telomerase levels are connected to human life expectancy represents an enormous challenge. Both the large variation in average telomere length between human individuals as well as the indirect role of telomeres in life expectancy represent major hurdles. Even if enough human embryos could be made available for study, telomere length measurements by either TRF or flow FISH would be unsuitable given the numbers of cells required for such studies. While novel techniques promise to overcome some of the limitations of current telomere length measurements (Lansdorp, 2022), further studies of the relation between telomere length and life expectancy are expected to strengthen correlations, not establish causality. For these reasons, the proof that higher levels of dyskerin and telomerase in early embryos are a major contributor to the sex difference in average human life expectancy is not expected in the near future.

## CONFLICT OF INTEREST

The author is a founding shareholder of Repeat Diagnostics Inc., a company specializing in clinical telomere length measurements since 2006.

## AUTHOR CONTRIBUTIONS

Peter Lansdorp is the sole author of this paper.

## Data Availability

Data sharing is not applicable to this article as no new data were created or analyzed in this study.
